# Generative Adversarial Network-Based Data Augmentation for Enhancing Wireless Physical Layer Authentication

**DOI:** 10.3390/s24020641

**Published:** 2024-01-19

**Authors:** Lamia Alhoraibi, Daniyal Alghazzawi, Reemah Alhebshi

**Affiliations:** Faculty of Computing and Information Technology, King Abdulaziz University, Jeddah 21589, Saudi Arabia; dghazzawi@kau.edu.sa (D.A.); ralhebshi@kau.edu.sa (R.A.)

**Keywords:** generative adversarial networks, wireless physical layer authentication, convolutional neural network

## Abstract

Wireless physical layer authentication has emerged as a promising approach to wireless security. The topic of wireless node classification and recognition has experienced significant advancements due to the rapid development of deep learning techniques. The potential of using deep learning to address wireless security issues should not be overlooked due to its considerable capabilities. Nevertheless, the utilization of this approach in the classification of wireless nodes is impeded by the lack of available datasets. In this study, we provide two models based on a data-driven approach. First, we used generative adversarial networks to design an automated model for data augmentation. Second, we applied a convolutional neural network to classify wireless nodes for a wireless physical layer authentication model. To verify the effectiveness of the proposed model, we assessed our results using an original dataset as a baseline and a generated synthetic dataset. The findings indicate an improvement of approximately 19% in classification accuracy rate.

## 1. Introduction

Ubiquitous technologies are experiencing significant growth, characterized by the development of several innovative systems, including smart vehicles, smart homes, smart cities and intelligent applications in industries and healthcare. Emerging ubiquitous technologies, such as the Internet of Things (IoT), are significantly increasing the number of devices used. Furthermore, the German online platform Statista Inc. expects 17 billion IoT devices to be actively deployed by 2030 [1].

Due to the openness of wireless networks, practically every wireless-receiving device within a given range can receive signals, which creates serious network security issues because both authorized and unauthorized users can use the communication channel. However, the preservation of integrity, confidentiality and availability presents significant challenges in the context of wireless networks [2]. As a result of the broadcasting nature of wireless communications, authentication is a crucial problem [3]. To prevent malicious users and only allow authorized users to access a wireless network, device identity authentication is essential [4].

To effectively address these challenges, it is important to employ robust and highly efficient techniques to mitigate the risk of device impersonation. These techniques must be stable, regardless of environmental conditions or device movements. Wireless physical layer authentication (WPLA) is a promising approach that offers a comprehensive framework for addressing security concerns in wireless communication. It is an approach for identifying wireless transmitters by analyzing the physical layer features of transmissions [5].

With the continual development of artificial intelligence (AI) technologies, machine learning (ML) and deep learning (DL) have grown to influence various aspects of people’s daily lives. With great results in numerous challenging cognitive experiments, DL computing has steadily risen to become the most used computational approach for ML. Significantly, DL has surpassed other well-known ML approaches in several fields due to its superior data analysis capabilities and accuracy.

Due to DL’s excellent classification capabilities, deep neural networks (DNNs) perform exceptionally well for WPLA. Baldini et al. [6] used convolutional neural network (CNN) and recurrence plot techniques to develop classification approaches for the physical layer authentication challenge. To identify different devices by utilizing distinctive radio frequency fingerprints (RFFs), Aminuddin et al. [7] presented a methodology based on a CNN to secure wireless transmissions in wireless local area networks. Liao et al. [8] adopted DNNs, CNNs and convolutional preprocessing neural networks to perform physical layer authentication in industrial wireless sensor networks. Furthermore, some research has examined the relationship between the number of hidden layers and authentication rate and has discovered that authentication rate improves as the number of hidden layers increases. In contrast, Ma et al. [9] used long short-term memory as an effective classifier to determine authorized and unauthorized users and increase detection efficiency and accuracy through simulations under varied channel conditions.

The primary objective of the WPLA approach is to identify wireless nodes effectively and accurately. The utilization of DL models in WPLA has demonstrated remarkable performance. However, DL models often require an enormous amount of labeled data to perform well. Consequently, this becomes particularly challenging when acquiring labeled data is time-consuming or costly, which raises concerns regarding retraining WPLA models whenever wireless nodes join or leave networks.

In this study, we suggest utilizing generative adversarial networks (GANs) [10] for data augmentation in a method that considers the real features of wireless nodes. The main focus is to leverage the enhanced learning capabilities of GANs to generate high-quality, labeled wireless node data using a limited amount of existing real data. Incorporating this enhanced dataset is expected to significantly enhance the classification accuracy of WPLA models.

The primary contributions of this study are summarized as follows:

- This study proposes a GAN–WPLA model for enhancing WPLA results, introducing a GAN architecture to generate a synthetic dataset by effectively learning the probability distribution of real data. To improve the diversity and reliability of the synthetic dataset, this study introduces different techniques to ensure the stability of the model during the training process;
- This study aims to improve and empirically establish the resilience of the WPLA model’s node classification performance. Moreover, this study conducts the training process using a CNN on a hybrid dataset containing real and synthetic data. The results demonstrate the beneficial synergy of integrating the GAN and WPLA models;
- This study uses a publicly available dataset to conduct the investigation. Further, this study endeavors to affirm the effectiveness of the proposed model for synthesizing high-quality labeled channel impulse response (CIR) datasets;
- This study defines the influence of several essential parameters on the performance of the GAN–WPLA model. Specifically, we systematically analyze the impact of the following factors: the number of wireless nodes, the signal-to-noise ratio (SNR) and the number of layers within the CNN classifier structure. This study offers new insights into how these factors interact with each other in CIR data synthesis and how that affects classification accuracy.

The remainder of this paper is organized as follows. Section 2 presents the background of GAN usage and Section 3 reviews related works. Section 4 introduces the methodology and proposed models, while Section 5 discusses the analysis of the results. Finally, the conclusion is presented in Section 6.

## 2. Background

This section provides an overview of the fundamental concepts related to GANs, aiming to provide the necessary knowledge to comprehend the subsequent sections. In the field of AI, generative models play an essential role. In 2014, Goodfellow et al. [10] presented a significant class of generative models known as GANs. They have been successful because of their ability to generate and adapt high-quality data in various fields. GANs are computational models utilized for data generation, which involves the creation of generative models that closely resemble real datasets. This is achieved by implementing adversarial games in which two players engage competitively. The two players represent a discriminator and a generator, both of which can employ the structure of the current DNN [11,12]. The generator takes Gaussian noise as input and creates an output that generates data that are similar to real data. The discriminator is commonly a probabilistic classifier designed to differentiate between real and generated data. The primary objective of the generator is to generate data that closely resemble real data, intending to deceive the discriminator. Conversely, the discriminator’s role is to accurately distinguish between real and generated data, regardless of the generator’s level of deception.

The procedure begins with the generator producing a random set of samples. To train the discriminator to distinguish the differences between these samples and another set of real samples, the samples are first fed into the discriminator. Once the differences have been found, the discriminator provides feedback to the generator. The generator depends on this feedback to enhance the generative process, producing instances that are more realistic [10]. In alternating stages, both neural networks are trained. Throughout the iterative process, the discriminator continuously enhances its ability to differentiate between real and generated samples, while the generator simultaneously improves its ability to generate highly realistic samples that can successfully deceive the discriminator.

**Figure 1:**
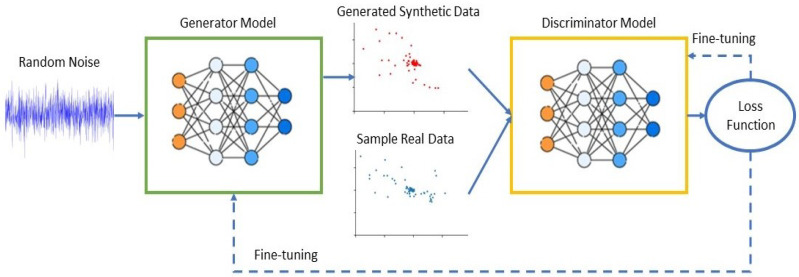
General workflow of the GAN architecture.

The fundamental concept of GANs is derived from the Nash equilibrium in the field of game theory [13]. To win the game, both players must continuously optimize their respective generation and discrimination abilities. The objective of an optimization process is to identify a Nash equilibrium between the two players. The general workflow of a GAN is shown in Figure 1. The main goal is to reach a condition in which the generator can reliably produce synthetic data that are indistinguishable from the input domain samples, while the discriminator is similarly incapable of distinguishing between the two types of data. When both models have been trained to the point where further improvement is impossible, it is sometimes said that the network has reached Nash equilibrium.

### 2.1. Architectures of GANs

Many efforts have recently been made to increase the performance of GANs by making various modifications to the original architecture. For instance, Arjovsky et al. [14] proposed the Wasserstein GAN (WGAN) and Radford et al. [12] introduced a set of network structures known as deep convolutional GANs (DCGANs), which enable the simultaneous training of deep convolutional generator and discriminator networks. Chen et al. [15] suggested the utilization of InfoGAN to effectively capture the mutual information that exists between a limited number of latent variables and observed data. Mirza et al. [16] designed conditional GANs (CGANs) by adding class conditionalities to generator and discriminator networks. In addition to previous adversarial autoencoders, networks consist of an “encoder” and “decoder” that learn to map data to internal latent representations.

### 2.2. Applications of GANs

GANs have many applications, including in fields such as computer vision and natural language processing, among others. Notably, image synthesis has been one of the most extensively researched domains within GAN application, with studies revealing the substantial potential of GANs in this context. For instance, the BEGAN model [17] has been a prominent illustration, showcasing the capacity of GANs to generate high-quality facial samples at resolutions of 128 × 128, which exhibit remarkable diversity.

Conversely, GANs have also been applied in the fields of speech and language processing. For instance, Li et al. [18] harnessed GANs to capture the contextual relevance of dialog and generate corresponding textual content. Furthermore, GANs have exhibited significant promise in text-to-image synthesis. Ku et al. [19] introduced TextControlGAN, a specialized GAN-based model for text-to-image synthesis tasks. In contrast to conventional GANs, TextControlGAN incorporates a neural network structure to effectively extract features from conditional textual descriptions.

Furthermore, GANs have also been applied within the realm of cybersecurity. For instance, Hu et al. [20] introduced a GAN-based algorithm called MalGAN, which was designed to generate adversarial malware samples. These adversarial malware samples can evade detection via black box machine learning-based models. In the same context, Chougule et al. [21] highlighted the vulnerabilities of controller area network (CAN) bus systems, particularly the lack of source verification for received messages, which make it susceptible to various attacks. To address the challenges of the limited data for training deep learning-based security mechanisms for the CAN protocol, they introduced a GAN method called SCAN-GAN for generating synthetic CAN data.

In the field of medicine, Brophy [22] generated synthetic electrocardiogram (ECG) signals that accurately represent the waveforms typically observed in patients. This accomplishment included successfully generating high-quality synthetic time series data while preserving the privacy and confidentiality of the training dataset. Hazra et al. [23] introduced the SynSigGAN model, which was designed to generate various types of synthetic biomedical signals. The generation of synthetic signals addresses concerns related to the confidentiality and accessibility of medical data. Such synthetic data can serve multiple purposes, including training medical students and machine learning models to advance and automate healthcare systems.

In a different field of GANs application, Li et al. [24] introduced a groundbreaking and integrated architecture known as ActivityGAN. This architecture was specifically designed for the effective generation of sensor-based data that emulate human physical activities. Their study involved the training of this novel architecture using a dataset of human activity information. The output generated by the generator, which comprises synthetic data, is subsequently evaluated using visualization methods. Additionally, Bui et al. [25] proposed a method that harnesses the capabilities of GANs to augment fault signals, effectively enriching the available dataset. This enhanced dataset has the potential to improve the accuracy of machine fault detection models during the training process.

## 3. Related Works

This section explores recent contributions in which GAN-based models have been applied within wireless networks. Several DL models have recently been implemented in this domain, but we focus on GAN-based models.

Davaslioglu and Sagduyu [26] introduced a new approach to spectrum augmentation using GANs. This approach effectively handles the inherent difficulties of applying ML classifiers in the cognitive radio domain. Generative adversarial learning is implemented to obtain and generate synthetic data samples, enhancing classifier accuracy via training data augmentation. Additionally, they demonstrated that the inclusion of high-fidelity training data in the retraining process of the classifier leads to improved accuracy in spectrum sensing. This improvement is comparable to the hypothetical scenario, where extra real data are available within the same spectrum environment. Gong et al. [27] proposed a framework for identifying specific emitters utilizing the InfoGAN approach. To enhance the quality of GANs, the proposed framework contains two additional inputs. The proposed framework involves the creation of an artificial RFF vector. This vector is generated using the histograms of bispectral information extracted from received signals. This synthetic RFF vector aims to improve the distinction between individual elements. Additionally, a structured multimodal latent vector is utilized. This latent vector incorporates prior knowledge of fading channel distributions and was designed to align with the characteristics of received signals. The proposed framework demonstrated an assessment score of 87% for the GAN generator. Truong and Yanushkevich [28] proposed an approach for synthesizing radar signals using GANs. Their research explored the application of GANs in the domain of one-dimensional radar signal data generation and augmentation, which has yet to be thoroughly studied. The approach intends to replicate simplified real scenarios in which suspects endeavor to obscure highly reflective objects underneath multiple layers of clothing. Training samples are used to train GANs to generate samples that closely match the training data distribution. This approach has presented encouraging outcomes in synthesizing indiscernible radar signal samples from training samples.

In the same context, Roy et al. [29] provided complete band spectrum simulations and emulation examples using GANs that are sufficiently realistic. In addition, they considered the need for signal synthesis to validate and show that such an approach was feasible, improve the algorithmic approach and quantify and prove its efficiency with current signal sets. Tang et al. [30] addressed the issue of limited labeled data. They looked into the potential of using GANs to effectively generate images to expand original datasets through data augmentation. The findings of this experiment suggest that the GAN-based data augmentation framework used in this study can potentially enhance CNN classification performance, resulting in accuracy improvement ranging from 0.1–6%. Castelli et al. [31] investigated using GANs to generate synthetic telecommunication data about Wi-Fi signal quality. Vanilla GANs and Wasserstein GAN architectures were employed in this study. According to the results of their experiment, both models can generate synthetic data whose distribution matches that of actual data. Additionally, they demonstrated that an ML classifier showed a limited ability to differentiate between actual and generated data, thus providing further evidence of the resilience of the GAN-based model.

Furthermore, He et al. [32] introduced a communication signal enhancement model to address environmental changes. They developed a model utilizing GANs, which includes an encoding–decoding structure based on convolutional layers. This model’s primary objective is to mitigate noise and interference in signals expeditiously. The experimental findings confirm that the proposed model provides greater efficacy in enhancing communication signals. Zhou et al. [33] developed a generic adversarial framework called the wireless signal enhancement GAN for wireless signal enhancement. Ablation studies were employed to demonstrate the value of each part of the objective’s function. Unlike the signal enhancement method, which learns noise distribution and interference characteristics and then removes them from the original signals, this generator learns the characteristics of signals in an adversarial manner. Patel et al. [34] suggested a data augmentation approach involving the utilization of conditional GANs for automatic modulation classification. Their objective was to utilize conditional GANs to produce high-quality labeled data using just a small quantity of initial data. This approach addresses the significant expenses and difficulties faced in acquiring wireless datasets. Through experimentation using an open-source dataset, they demonstrated that the suggested data augmentation approach can significantly enhance the performance of automatic modulation classification models.

In the fields of physical layer authentication and signal processing, the learning abilities of GANs have not yet been extensively adopted. The primary objective of our research was to exploit GANs to augment datasets and improve the training of classifiers used in physical layer authentication. This objective was prioritized due to the remarkable capability of GANs to accurately estimate complex probability distributions, even when provided with limited amounts of data.

## 4. Research Methodology

### 4.1. GAN–WPLA Model Architecture

**Figure 2:**
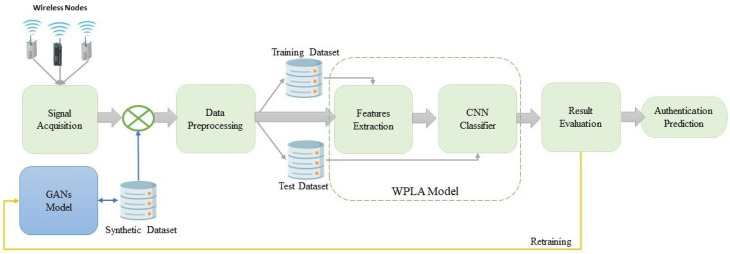
Architecture of the GAN–WPLA model.

This section presents the general architecture for the proposed GAN–WPLA model. As represented in Figure 2, we propose two models grounded in a data-driven approach. The GAN model is implemented as an efficient data augmentation method to enhance the classification performance of the WPLA model. The first step is to obtain reliable wireless signal samples before implementing the WPLA model. Due to the unpredictability of the wireless environment, the available datasets for training are limited as acquiring large datasets is considered a time-consuming procedure. We introduce a GAN model for augmenting data that exploits the unique characteristics of wireless CIRs. The next step is data preprocessing and following that, we store the samples by properly splitting them into two datasets. Subsequently, we proceed with the training of the WPLA model. Ultimately, the effectiveness of the WPLA model is evaluated and in cases where improvements are required, a retraining query is initiated.

#### 4.1.1. Data Acquisition

The foundation of a powerful DNN model is a sufficiently large training dataset. Therefore, we must first determine how wireless signals are represented before we start the data augmentation method. In wireless node identification, the goal is to detect the presence of transmitters from channel state information (CSI). The received wireless signal *r*(*t*) is usually given by Equation (1).

(1) r(t)=h(t)×s(t)+n(t)

where *s*(*t*) is the transmitted signal, *n*(*t*) is the additive white Gaussian noise and *h*(*t*) is the CIR.

The CIR dataset used in this study was created by the National Institute of Standards and Technology (NIST) [35]. The CIRs were obtained at a standard industrial site: a machine shop. The machine shop is an indoor setting with outer dimensions of approximately 12 m × 15 m and a ceiling height of approximately 7.6 m. The distances between the transmitter and receiver did not exceed 50 m. The CIRs were captured while the receiving equipment was moved from one acquisition point to another during CSI measurements, so each record represents a different position. The maximum distance between each acquisition point was 1 m. The center frequency of the transmitter and receiver was 5.4 GHz. In addition, the transmitter constantly transmitted a PN sequence modulated with a binary phased-shift keying signal of length 2047 while the receiver upsampled by a factor of 4. Thus, the CIR in each record is an 8188 × 1 complex vector in the time domain. All raw data are stored in a complex-valued, traditional in-phase (*I*) and quadrature (*Q*) signal representation. The set of *I/Q* vectors with lengths ranging from 0 to (n − 1) represent the CIRs of the signals and the time stability characteristics of the signals, denoted as *T*, can be expressed as follows:

$$r_{raw}={\left[r_{I}\left[0\right],r_{I}\left[1\right],\ldots r_{I}\left[n-1\right]\right]}^{T}+j{\left[r_{Q}\left[0\right],r_{Q}\left[1\right],\ldots r_{Q}\left[n-1\right]\right]}^{T}$$

#### 4.1.2. GAN Model-Based Data Augmentation

**Figure 3:**
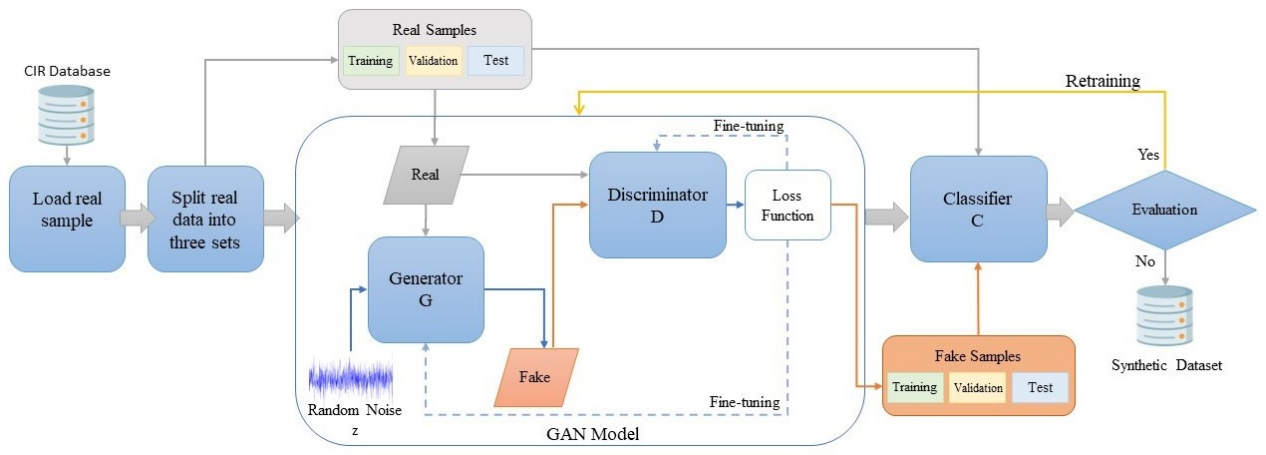
Proposed GAN model.

DL approaches show exceptional classification capabilities, yet their effectiveness diminishes when confronted with relatively limited datasets. Small datasets are frequently encountered in wireless communication because of the temporal correlation of wireless signals during propagation. The overall structure of the proposed GAN model is shown in Figure 3.

GANs represent ML approaches that are designed to generate samples that closely resemble real data, with the objective of making them indistinguishable [10]. GAN models consist of two neural networks: a generation network Generator (*G*) and discriminator network Discriminator (*D*).The primary purpose of the generator is to imitate the distribution of real signals and generate fake signals that are similar, where *G*(*z*) is the generator output and *z* is the noise input to the generator with a model distribution of *Pz*. It is important to note that the generator learns the data distributions of real datasets rather than memorizing input–output pairs. The discriminator is a standard binary classifier, giving a value of 0 to signals that are generated and a value of 1 to signals from the real dataset. The purpose of the discriminator is to identify differences between fake data *G*(*z*) and real data *r*, for which r∈realdataset has a distribution *Pdata*.

In this model, two distinct loss functions are taken into consideration, with one applied for the generator and the other for the discriminator. However, the two loss functions are derived from a comparison of the two probability distributions, one produced by the generator *Pz* and the other from the real dataset Pdata. The model can be considered a min–max game, in which the generator’s goal is to minimize the function by generating samples that are indistinguishable from real ones, while the discriminator’s goal is to maximize the function. The formula for this learning process is as follows:

(2) $$minG\;maxD\;V\left(D,G\right)=E_{r∼P_{data}\left(r\right)}\left[\log\left(D\left(r\right)\right)\right]+E_{z∼P_{z}\left(z\right)}\left[\log(1-D\left(G\left(z\right)\right)\right]$$

where *Er* is the expected value over all real data, *D*(*r*) is the discriminator’s estimate of the probability of real data, *Ez* is the expected value over all of the *G*(*z*) and *D*(*G*(*z*)) is the discriminator’s estimate of the probability that a *G*(*z*) is real. As shown in the formula, the evaluation of the discriminator’s ability to accurately identify the samples involves the use of two different terms. The first measure, denoted as $E_{r∼P_{data}}\left[\log\left(D\left(r\right)\right)\right]$, evaluates the ability of the model to accurately identify real data. In contrast, the second measure, $E_{z∼P_{z}}\left[\log(1-D\left(G\left(z\right)\right)\right]$, evaluates the model’s ability to distinguish fake data.

Consequently, during the training of the generator, the term that considers real data is ignored and the generator parameters are updated by only considering the loss function values of the fake data. However, to ensure that the fake data are closer to the distribution of the real data, the loss function of the *G* is defined as follows:

(3) $$L_{G}=\log\left[1-D\left(G\left(z\right);\theta_{d}\right)\right]$$

On the other hand, the discriminator parameters are updated by considering the loss function values of both the real and fake data. The discriminator loss function is as follows:

(4) $$L_{D}=E_{r∼P_{data}\left(r\right)}\left[\log\left(D\left(r;\theta_{d}\right)\right)+\log\left(1-D\left(G\left(z\right);\theta_{d}\right)\right)\right]$$

Therefore, the best result for the discriminator is *D*(*r*) = 1 and *D*(*G*(*z*)) = 0. The competition terminates if the discriminator is unable to distinguish between the real and fake data and the generator is unable to enhance the quality of the data, resulting in the achievement of a state termed “Nash equilibrium”.

However, GANs pose specific challenges [36], including the nonconvergence result. To address this challenge, we follow the ACGAN framework [37]. In contrast, we add Classifier (*C*), which has the same structure as a discriminator. So, when the GAN model achieves the Nash equilibrium state and to ensure that the fake sample dataset converges with the real sample dataset, we train the classifier. The training goal of the classifier is to be used as a surrogate model to simulate the function of the model discriminator. The classifier’s primary function is to evaluate the degree of similarity between the fake samples generated by the GAN model and the real samples. Following an evaluation process, the most convergent samples are aggregated into a synthetic dataset. This synthetic dataset is subsequently employed in the training of the WPLA model, ultimately enhancing the robustness of the authentication process.

#### 4.1.3. Data Preprocessing

In this work, we examine the utilization of the fast Fourier transform (FFT) function to convert CIRs into discrete representations known as the channel frequency responses (CFRs). The FFT function is employed to transform the raw time domain data into its corresponding frequency domain representations. The raw data *r* of the CIRs are kept in a format that consists of complex numbers. To simplify the representations, these complex numbers are transformed into two sets of real-valued data vectors. This transformation allows us to express the data in a form that is easier to understand, as follows:

$$r={\left[\begin{array}{c} r_{I}\\ r_{Q} \end{array}\right]}^{T}$$

The translation to the frequency domain is performed first by obtaining the amplitude using Equation (5).

(5) $$Ai=\sqrt{Ii^{2}+Qi^{2}}$$

where *Ai* denotes the amplitude of *Ii* and *Qi*, which represent the *i*-th data in *r* data vectors.

Next, we execute the FFT function. The Fourier transform is a mathematical operation that relates a function in the time domain to its representation in the frequency domain, where the magnitude refers to the frequency. The Fourier transform can be applied to a variety of domains, although the time domain is often considered the original function. The discrete Fourier transform can be performed in the time domain by applying the following equation:

(6) $$X\left(i\right)=\frac{1}{N}\sum_{n=0}^{N-1}x\left(n\right)\cdot e^{-\frac{j2\pi ni}{N}}$$

Here, X(i) characterizes the frequency content of the time samples x(n) associated with the original signal r(t), where *N* representing the size of the domain for the results of the sum of value *n*.

Finally, to obtain the CFR vectors, we need to repeat Equation (5) and obtain the amplitude of the complex vectors. After data preprocessing, we construct training and test datasets, where each datasets is composed of:

$$\begin{array}{c} Datasets\{X\;set,L\;set\} \end{array}$$

where *X set* is the CFR vectors of each node and *L set* is the category label corresponding to the CFR vectors of the wireless node transmitter.

#### 4.1.4. WPLA Model

**Figure 4:**
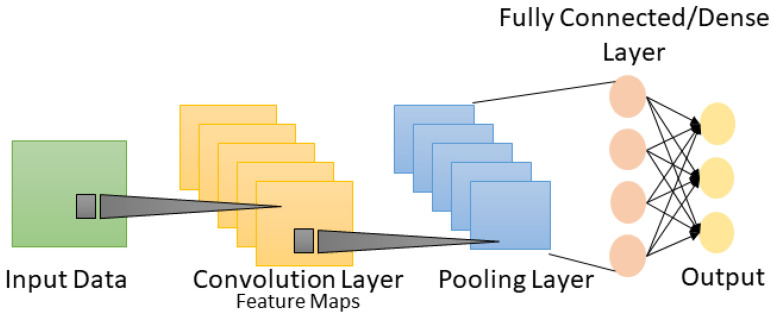
Basic architecture of a CNN.

The identification of wireless nodes by the WPLA model can be considered as a mapping function from CFR vectors to categories of wireless node transmitters. Different DNN architectures can be implemented as classifiers. We chose a CNN architecture for this work as CNNs have been commonly applied in various domains over the past few decades due to their significant feature extraction ability. CNNs are typically designed for processing data with known grid-like structures, such as time series data, which can be seen as one-dimensional structures, as well as the two-dimensional (2D) structures of pixels in image data and the three-dimensional structures of videos [11]. The CNN architecture comprises a visible layer that indicates the multidimensional input data and several hidden layers that extract features based on spatial distances. The basic architecture of a CNN is shown in Figure 4. The convolutional layer acts as the most essential part of the CNN architecture. Each convolutional layer comprises several filters that work together to create a massive convolution of the input and feature maps. The learning algorithm trains the filters through a backpropagation training mechanism, often with a multidimensional array of parameters [11]. The filter sizes are chosen based on the input data sizes to have a suitable amount of abstraction that can be created at the correct scale.

For instance, a convolutional layer convolves over a 2D input (*x*) using 2D filters (*k*) to extract features, which is represented as follows:

(7) $${\left(x*k\right)}_{m,n}=x\left[m,n\right]*k\left[m,n\right]=\sum_{i}\sum_{j}x\left[i,j\right]\cdot k\left[m-i\right]\left[n-j\right]$$

After the convolution, a bias term *b* and a pointwise nonlinearity *f* are utilized to create a feature map at the filter output. The feature map is created using filters based on the input *x* and weights *w*, as follows:

(8) $$k_{m,n}=f\left({\left(x*W\right)}_{mn}+b\right)$$

The convolutional layer’s outputs are activated using a nonlinear activation function. Different types of nonlinear functions exist, such as the sigmoid, tanh and rectified linear unit (ReLU). The most common activation function of ReLU is represented as follows:

(9) f(x)=max(0,x)

A pooling layer is applied after a convolutional layer to perform a downsampling operation that lowers the in-plane dimensionality of the feature maps, introduces translation invariance to minor shifts and distortions and reduces the number of ensuing learnable parameters. The most fundamental part of the CNN architecture is the fully connected layer, which consists of fully linked neurons with all feature maps from the last layer. As a result, these layers are often known as dense layers. The final layer is a softmax classifier, which uses the following formula to calculate the posterior probability of each class label across *n* classes:

(10) $$S\left(y_{i}\right)=\frac{e^{y_{i}}}{\sum_{j=1}^{n}e^{y_{j}}},i=1,\ldots n$$

#### 4.1.5. Performance Evaluation Metrics

To assess the effectiveness of the data augmentation GAN-based model, we split the process of evaluation into two parts: qualitative and quantitative. The qualitative assessment of the GAN model relied on human visual assessment through the examination of the generated samples. Due to the need for an appropriate objective evaluation metric, this qualitative evaluation alone could not be considered a comprehensive assessment of the GAN model performance. In contrast, quantitative assessments cover several statistical measures used for time series analysis, such as error metrics. Error metrics are frequently employed for the evaluation of time series data. In this study, the mean absolute error (MAE) was utilized to assess the accuracy of the prediction results. The MAE shows the closeness between the actual and predicted values. MAE is calculated using the formula given in Equation (11).

(11) $$MAE=\frac{1}{N}\sum_{i=1}^{N}\left|y_{i}-{\hat{y}}_{i}\right|$$

where *N* is the number of values predicted by the model of the dataset used for prediction purposes, *y* is the set of actual values used as labels and $\hat{y}$ is the set of values predicted by the discriminator in the GAN model.

On the other hand, the performance of the WPLA model was evaluated using a number of performance indicators, including precision, recall and accuracy. The definitions of these metrics are provided in the next section. The metrics are defined by true positives (*TPs*), true negatives (*TNs*), false positives (*FPs*) and false negatives (*FNs*). The ratio of the classifier to the correct prediction of a node class is measured by the accuracy, as shown in Equation (12).

(12) $$Accuracy=\frac{\left(TP+TN\right)}{\left(TP+FN+FP+TN\right)}$$

Precision refers to the ratio of all positive predicted classes that are positive and correct. Equation (13) is used to compute precision.

(13) $$Precision=\frac{TP}{\left(TP+FP\right)}$$

Recall, also known as sensitivity, refers to the proportion of a model’s predicted positive and correct classes to the total number of actual positive classes. To estimate recall, we use Equation (14).

(14) $$Recall=\frac{TP}{\left(TP+FN\right)}$$

### 4.2. GAN–WPLA Model Training

This section introduces the optimized parameter configuration for the GAN–WPLA model. We present the optimal parameter combinations, as detailed in Table 1, Table 2 and Table 3. These tables encompass essential information, such as the number of filters, kernel size and activation function for each layer, as well as the total number of parameters for all layers. It is important to emphasize that the fine-tuning of these parameters significantly influences the speed and efficiency of the learning process, depending on the specific characteristics of the acquired data. Additionally, it is worth noting that each network’s structure is designed to meet particular requirements.

#### 4.2.1. Generator Structure

Within the GAN model, synthetic samples are created by utilizing latent variables and predicted category labels. A comprehensive depiction of the architectural characteristics of the generator can be found in Table 1. The first step in generating fake data is to feed the generator with a random number with a shape (1, 2047) that takes one point in the latent space. The following layer in the network is the dense layer, with 16 neurons, and LeakyReLU is used as the activation function with a 0.1 learning rate. This is followed by the reshape layer. We then apply transpose convolutional (Conv2Transpose) layers twice to double the input size with a kernel size of (3, 3). The last layer is a convolutional layer with one neuron. As is best practice for activation functions, we use hyperbolic tangent (tanh) activation in the transpose convolutional layers and the last convolutional layer. Finally, the output shape of the generated I/Q victor is (2, 8188, 1).

#### 4.2.2. Discriminator Structure

On the other hand, the discriminator network is shown in Table 2. The input tensor for the first layer is (1, 2, 8188), which receives r as the input data. The network typically uses two convolutional layers, with 256 and 128 neurons. LeakyReLU is also used as the activation function with a learning rate of 0.2. We apply a flattened layer to reshape the multidimensional output from the preceding convolutional layers into a one-dimensional vector. In addition, we use a dropout layer at a rate of 0.4 to prevent overfitting. The last layer is dense and only has one node to determine whether the data are real or fake. The activation function in the last layer is sigmoid. As we mentioned earlier, the classifier in the GAN model has the same structure as the discriminator.

#### 4.2.3. CNN Classifier Structure

In this section, we present the standard CNN network architecture used for the WPLA classifier responsible for CIR feature extraction. The structure of the CNN classifier is shown in Table 3. Moreover, we describe the visible and hidden layers of the proposed CNN model architecture. In the visible layer, we start by loading the CFR input samples. Next, we reshape the samples in the training and test sets to have a fixed size of (1, 1, 8188). The hidden layers consist of two convolutional layers. The first convolutional layer contains 256 filters, followed by a dropout layer. The second layer is a convolutional layer composed of 128 units, followed by a dropout layer. The combination of dropout regularization and the max norm has demonstrated excellent performance in preventing overfitting. This is followed by a flattening layer. The final layers in the CNN architecture are dense layers, which include neurons that are fully connected with all feature maps in the convolutional layers. The first dense layer contains 64 neurons and ReLU activation functions are applied to accelerate convergence during the training process. Finally, the last dense layer operates softmax activation to perform node classification.

To reduce the effect of the nonstationarity of wireless signal data, which gradually shifts in distribution over time, the proposed GAN–WPLA model offers an effective method to make the WPLA model more resilient by implementing the retraining process. This approach involves the incorporation of a training dataset that encompasses both real and updated synthetic samples. Retraining the WPLA model using this expanded dataset aims to enhance the training process, creating a more robust model.

## 5. Experiment Results and Analysis

We used the channel information dataset from a real industrial environment collected by the NIST [35] to illustrate our experimental analysis. This study aimed to assess the effectiveness of our GAN-based data augmentation model in enhancing a WPLA model based on a CNN architecture in wireless communication environments. We present an overview of the implementation of these models in Algorithms 1 and 2, respectively.
**Algorithm 1:** Pseudocode of the GAN model training process.
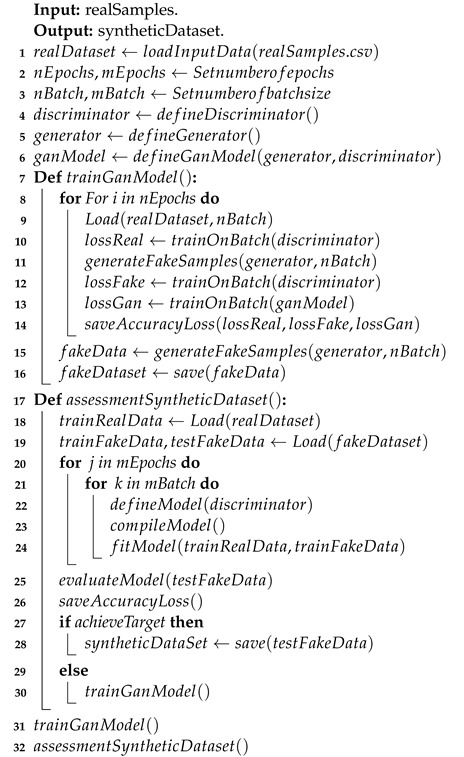


**Algorithm 2:** Pseudocode of the GAN–WPLA model training process.

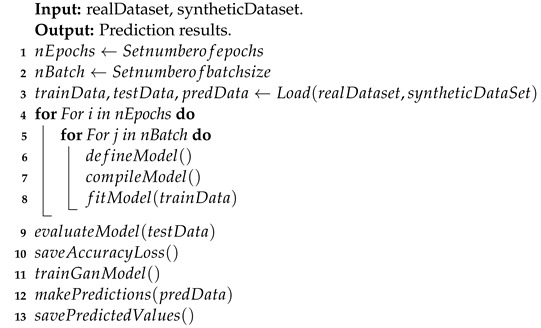



### 5.1. Training Implementation Details

The proposed models were implemented using Python 3.10.9, Keras library [38] and TensorFlow [39]. It is imperative to emphasize that the precise resource prerequisites are contingent upon the desired level of performance. Executing the GAN–WPLA model can incur considerable computational expenses.

Furthermore, the training of the proposed models was conducted on the Aziz supercomputer [40]. This computational facility comprises 496 computing nodes with approximately 12,000 Intel^®^ central processing unit cores. Additionally, the supercomputer features two nodes equipped with NVIDIA Tesla K20^®^ graphical processing units and two nodes outfitted with Intel^®^ Xeon-Phi accelerators. The collective RAM capacity across the system is 96 GB and the operating system in use is Linux version 3.10.0-1160.el7.x86_64. On the other hand, to achieve minimum acceptable performance, a workstation equipped with an NVIDIA graphics card and a high-performance CPU, such as Intel Core i7, paired with 16 GB of RAM is deemed requisite.

In Table 4, we provide an overview of the diverse hyperparameters employed in the configuration of the GAN–WPLA model, such as loss function, optimizer, epoch count, learning rate, batch size and dropout rate.

Furthermore, we allocated three datasets from the assembled database for the training, validation and testing of the GAN–WPLA model. The training dataset comprised 70% of the data, the validation dataset encompassed 10% and the test dataset involved 20%. In addition to the data, as mentioned earlier in the allocation, we applied the ’validation_split’ parameter within the Keras.fit() function for the collected dataset. This step was taken to ensure the comprehensive utilization of all collected data during the training and testing phases, thereby preventing overfitting during the training process.

### 5.2. GAN Model Analysis

The training process of the GAN model was executed sequentially on generator and discriminator networks, with a total dataset consisting of 1300 samples, each with 8188 data points representing a dataset of three industrial wireless nodes. The training started with the generator network, creating a synthetic I/Q vector using random seeds. The combination of real and generated I/Q vectors was used as input data to train the discriminator network. The loss was determined for each network using the outcomes provided by the discriminator. We adopted the Adam optimizer [41] to optimize the discriminator network parameters, with a learning rate of 0.0002. On the other hand, we applied RMSprop to optimize the generator network. The parameter epoch was set to 50 and the batch size was set to 128.

As previously indicated, GANs face challenges, including nonconvergence issues. Additionally, the case of mode collapse represents another considerable challenge. We applied different DL techniques to refine the training process. Accordingly, in the GAN model training, we monitored loss results and continuously adjusted the parameters to reach the optimal GAN structure, identify issues early and reduce the possibility of collapse. Therefore, the first parameter applied was the choice of MAE as a loss function in the generator. The main objective was to generate data as close to real data as possible. Using MAE encouraged the generator to produce I/Q vectors closer to the real data regarding feature-level similarity. The second parameter activation function, the I/Q vector distribution in the real dataset, was between —1 and 1. Consequently, we considered the tanh activation function to be an effective parameter in our situation, where we wanted the generated fake samples to be centered around zero. To mitigate the vanishing gradient problem, we applied Leaky ReLU to the discriminator.

**Figure 5:**
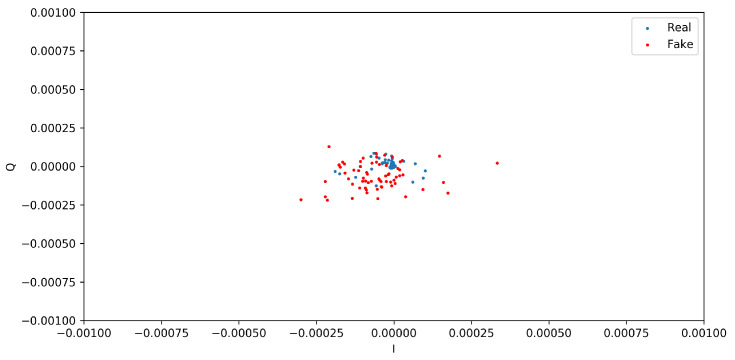
I/Q vector distributions based on the GAN model.

**Figure 6:**
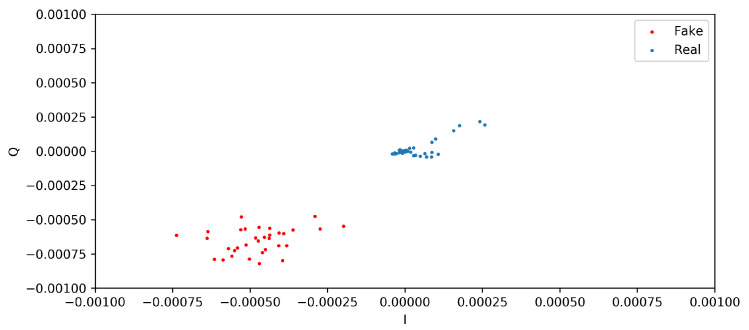
Instability of I/Q vector distributions based on the GAN model.

This section presents the two distinct qualitative and quantitative methods used for assessing the quality and similarity of the generated and real I/Q vectors. From the qualitative analysis, the scatter diagrams in Figure 5 and Figure 6 show the distributions of I/Q vectors based on the GAN model. Indeed, due to the limited availability of public CIR datasets of wireless nodes, it was necessary to create synthetic CIR samples by employing a GAN in conjunction with existing real samples. Therefore, generating the desired I/Q vector distributions presented a challenging task. The red dots in the figures denote the synthetic I/Q values, while the blue dots denote the real I/Q values. From Figure 5, we can observe that the two categories of I/Q values show overlapping distributions. Nevertheless, the GAN model sometimes showed instability throughout the training process. Figure 6 shows that the synthetic samples made by the GAN model were different from the real samples. The model could replicate the association between the synthetic I/Q vectors and the real ones in a broad sense; however, some situations were rejected due to the noncompliance of the synthetic generated I/Q vectors with the expected standards.

**Figure 7:**
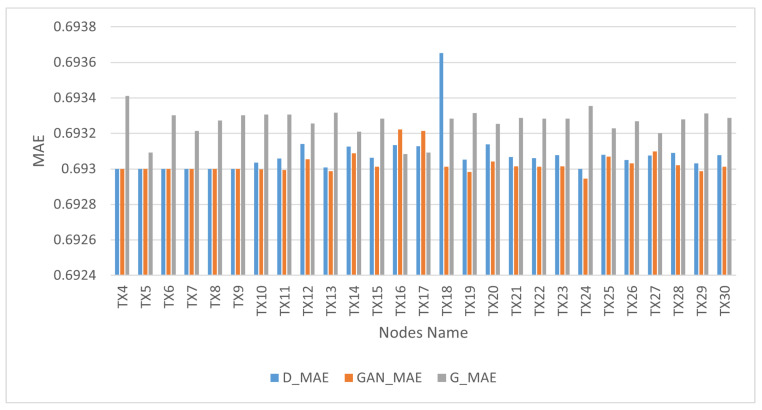
Overall GAN model performance versus nodes.

In the context of the quantitative analysis, MAE was utilized as a metric to assess the extent to which the distributions of the synthetic samples served as reliable approximations of the real distributions. We considered the sigmoid cross-entropy loss function when we used MAE in the training process because it can indicate the stability of generated and real data. The bins in Figure 7 illustrate the performance outcomes of the GAN network, discriminator and generator. The GAN network’s performance is represented by orange bins, the discriminator’s performance by blue bins and the generator’s performance by gray bins. The GAN model demonstrated the ability to reach equilibrium between the generator and discriminator networks, as evidenced by the lowest difference in loss degree throughout training of between 0.0001 and 0.0004.

### 5.3. GAN–WPLA Model Analysis

**Figure 8:**
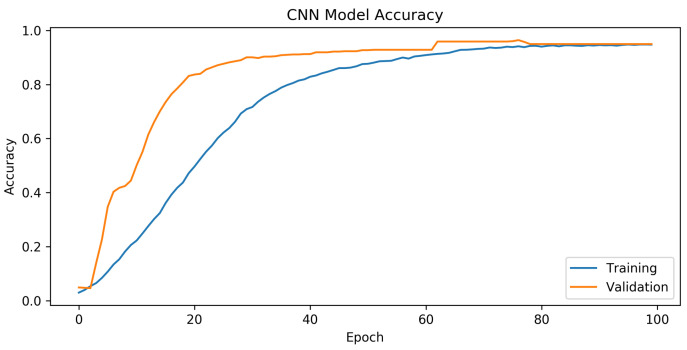
Accuracy over epochs for training the GAN–WPLA model.

**Figure 9:**
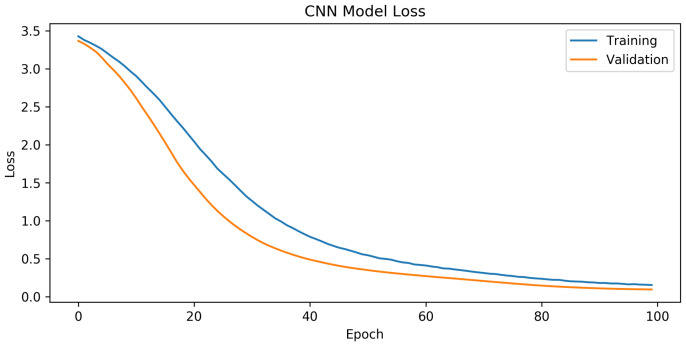
Cross-entropy loss over epochs for the GAN–WPLA model.

The analysis of the GAN–WPLA model’s performance was based on validating the satisfied CNN architecture classification of wireless nodes. To train the proposed model, we used datasets generated from the GAN model, which consisted of 18,850 samples, each with 8188 data points. We then divided it into two sets: the training set, which contained 15,080 (80%) samples, and the test set, which contained 3770 (20%) samples. During the experiments, we divided the training set into training and validation batches at a ratio of 7:1. In addition, we optimized the model parameters by adopting the Adam optimizer, with a learning rate of 0.001. The parameter training epoch was set to 100 and the batch size was set to 15,000. Category cross-entropy was adopted as the loss function to validate the model’s performance. In Figure 8 and Figure 9, the training graphics of the proposed model are presented.

According to the results presented in Figure 8, the CNN design showed exceptional performance. The mean training accuracy rate obtained was 73.3%, with the highest recorded rate reaching 94.8% across the epoch range running from 1 to 100. Based on the findings in Figure 9, it can be concluded that in the last part of the graph, there is a noticeable convergence of the training and validation losses, indicating the successful training of the CNN network. The model demonstrated a satisfactory configuration, as there was no evidence of either overfitting or underfitting.

**Figure 10:**
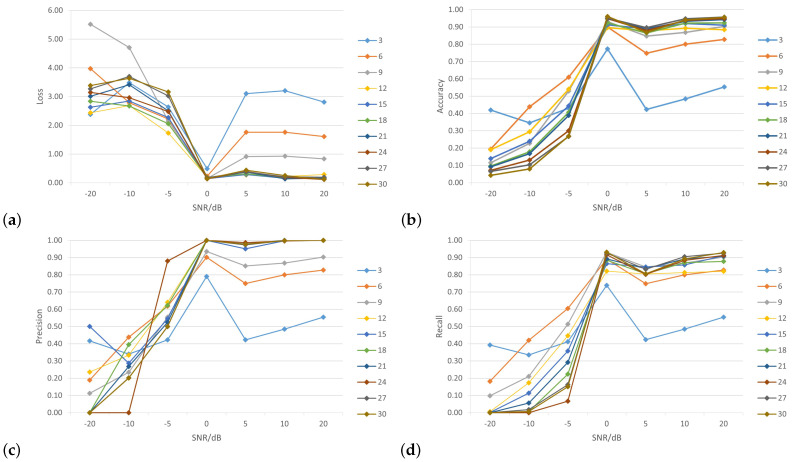
Overall classification results versus SNRs with different numbers of nodes: (**a**) loss; (**b**) accuracy; (**c**) precision; (**d**) recall.

Next, we present a comparison of the accuracy rate, loss, precision and recall of the proposed model after including different numbers of samples generated by the GAN model. To evaluate the robustness of the classification, Gaussian white noise was added to the generated data. This noise replicates the channel estimation error that develops throughout the channel estimation procedure. Figure 10 shows the impacts of different wireless nodes to SNR levels (0 dB, —5 dB, —10 dB, —20 dB, 5 dB, 10 dB and 20 dB) on the performance of the WPLA model.

In Figure 10a, the horizontal axis represents the number of SNR levels while the vertical axis represents the value of the loss function. As SNR levels increased, the value of the loss function decreased. Additionally, the loss function value decreased more as the number of nodes increased, although it is noticeable that the loss function value was still high with fewer nodes (such as 3, 6 and 9). Figure 10b shows the relationship between the overall classification accuracy and SNR for varying numbers of wireless nodes, where the CNN was trained using the training-generated dataset to assess the effectiveness of our proposed model. In general, the accuracy of classification demonstrated an upward trend with an increase in SNR. When the SNR levels increased from 20 dB to 0 dB, there was a significant increase in classification accuracy. The initial findings indicated that at SNR levels of 0 dB and 20 dB, the average test accuracy surpassed 95% while the precision rate reached 100%. Therefore, the proposed model can achieve both accuracy and precision. When the SNR was less than 10 dB, the energy of the noise was significantly greater than the energy of the signal. In this scenario, the extraction of CIRs from background noise is a significant challenge for all existing techniques. The model lacked classification capability, resulting in an average classification accuracy of approximately 14%. Furthermore, the precision and recall results of the classification are shown in Figure 10c,d. It is clear from the figures that as the number of nodes and SNR levels increased, the model showed excellent performance, with a precision rate of 100% and a recall rate of 93%.

**Figure 11:**
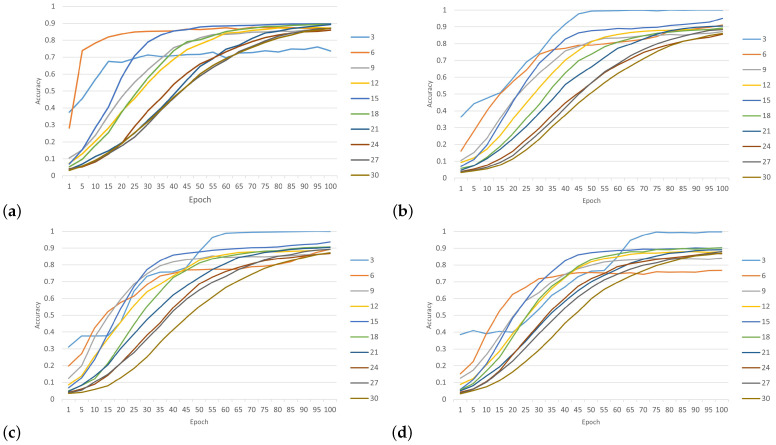
Accuracy of classification with different numbers of nodes and SNR levels: (**a**) SNR = 0 dB; (**b**) SNR = 5 dB; (**c**) SNR = 10 dB; (**d**) SNR = 20 dB.

The proposed model was tested with 30 wireless nodes at four SNR levels (0 dB, 5 dB, 10 dB and 20 dB). Figure 11 shows the performance training of the CNN network. As the SNR increased, the extraction of the CIRs became more feasible, leading to a continuous improvement in training accuracy. When the SNR was 0 dB, the power of the noise was equivalent to the power of the signal. This scenario could be interpreted as occurring within highly noisy environments. In the context of simulated environments, it is unlikely that classification accuracy would experience a substantial decrease. The impact of environmental noise on discrimination was significant. When the model classified three nodes, we can see that when SNR was 0 dB, the accuracy rate was 76% and began to increase significantly until reaching 100% with SNR levels of 5 dB, 10 dB and 20 dB. On the other hand, when the model classified more nodes, we can see that the accuracy rate slightly improved in SNR environments of 5 dB, 10 dB and 20 dB. However, both SNR levels of 5 dB and 10 dB showed better classification performance. In addition, we found that when the model classified 15 nodes, the accuracy rate was 95% for SNR levels of under 5 dB, but when the number of nodes was greater than 15 in the SNR environment of 20 dB, the accuracy rate of classification increased relatively. Overall, the proposed model demonstrated a higher level of accuracy in identifying the source nodes of received CIR signals.

**Figure 12:**
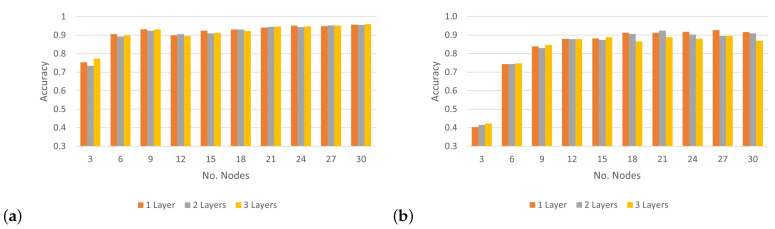

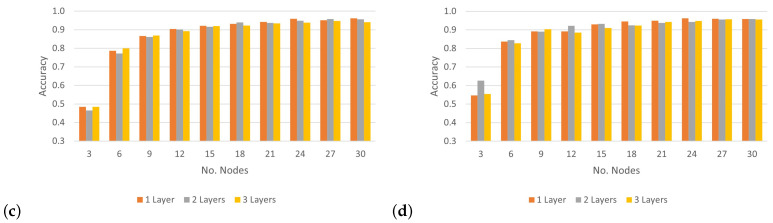
Accuracy of classifying numbers of nodes using numbers of convolutional layers at different SNR levels: (**a**) SNR = 0 dB; (**b**) SNR = 5 dB; (**c**) SNR = 10 dB; (**d**) SNR = 20 dB.

To analyze the impact of applying various numbers of hidden layers on the CNN’s accuracy rate and determine the optimal layers for classifying 30 wireless nodes, Figure 12 shows the results of the accuracy rate of the proposed model, which uses three convolutional layers at different SNR levels. Based on the results in Figure 12a, under the condition of an SNR level of 0 dB, the CNN architecture could achieve more than 90% accuracy when classifying 15 nodes or more by applying different numbers of convolutional layers. Furthermore, when the model tried to classify three nodes, we can see the best accuracy was recorded under an SNR of 0 dB. However, in the case of an SNR of 5 dB, the performance of the model with a different number of convolutional layers recorded a low classification accuracy, with an average rate of 81.8%. Following the model, performance with SNRs of 10 dB and 20 dB was similar and considered better than that with an SNR of 5 dB, with an average rate of more than 87%. As a result, there were no significant differences in the performance of the CNN architecture when increasing the number of layers. However, notable differences were apparent when the number of nodes was relatively small. Based on these experiments, the best number of convolutional layers for the CNN architecture was one layer with a suitable number of output filters, which further improved accuracy without increasing computation.

In summary, experimental validation showed that the GAN–WPLA model worked well to strengthen the WPLA model in relation to complex and changing channel information.

## 6. Conclusions

Wireless networks are essential for providing reliable communication across ubiquitous technologies. The inherent wireless nature of these networks requires advanced intelligent techniques to effectively manage and enhance security risks. DL has become increasingly popular in security due to its ability to analyze large amounts of data and identify complex patterns. Although DL-based models can be effective in security applications, they require considerable annotated training datasets. Gathering new data is recognized as a costly and challenging task. In this study, we proposed a GAN–WPLA model that leverages generative adversarial learning to learn and generate high-quality synthetic CIR samples that improve classifier accuracy. A GAN-based data augmentation approach was implemented to enhance the WPLA model. Through experiments, the proposed data augmentation approach was shown to greatly benefit from CNN-based wireless node classification. The experimental results indicated that the model could generate synthetic data that matched real distributions. In addition, the results demonstrated that GAN-based data augmentation could improve CNN classification and that the proposed model obtained a 19% increase in accuracy rate. In future work, we intend to investigate the effectiveness of the GAN–WPLA model for detection systems. Additionally, we will examine the performance of other DL architectures within the context of WPLA.

## Figures and Tables

**Table 1:** Proposed generator structure.

Layer Type	Input Size	Parameters	Activation Function
Input Shape	1 × 2047	None	None
Dense Layer	1 × 2047	16 neurons	LeakyReLU
Reshape Layer	1 × 2047 × 16	None	None
Transpose Convolutional Layer	1 × 2047 × 16	32 neurons; 3 × 3 kernel size; 2 × 2 strides	tanh
Transpose Convolutional Layer	2 × 4094 × 32	64 neurons; 3 × 3 kernel size; 1 × 2 strides	tanh
Convolutional Layer	2 × 8188 × 64	1 neuron; 1 × 2047 kernel size	tanh

**Table 2:** Proposed discriminator structure.

Layer Type	Input Size	Parameters	Activation Function
Input Shape	1 × 2 × 8188	None	None
Reshape Layer	1 × 2 × 8188 × 1	None	None
Convolutional Layer	1 × 2 × 8188 × 1	256 neurons; 3 × 3 kernel size; 2 × 2 strides	LeakyReLU
Convolutional Layer	1 × 1 × 4094 × 256	128 neurons; 3 × 3 kernel size; 2 × 2 strides	LeakyReLU
Flatten	1 × 1 × 4094 × 256	None	None
Dropout	262,016	0.4 rate	None
Dense Layer	1	1 neuron	sigmoid

**Table 3:** Proposed CNN classifier structure.

Layer Type	Input Size	Parameters	Activation Function
Input Shape	1 × 8188	None	None
Reshape Layer	1 × 1 × 8188	None	None
Convolutional Layer	1 × 1 × 256	256 neurons; 1 × 3 kernel size; glorot uniform kernel initializer	ReLU
Dropout	1 × 1 × 256	0.4 rate	None
Convolutional Layer	1 × 1 × 128	128 neurons; 1 × 3 kernel size; glorot uniform kernel initializer	ReLU
Dropout	1 × 1 × 128	0.4 rate	None
Flatten	128	None	None
Dense Layer	128	64 neurons; he normal kernel initializer	ReLU
Dense Layer	64	30 neurons	softmax

**Table 4:** Hyperparameters used in GAN–WPLA model training.

Hyperparameter	Generator	Discriminator	CNN Classifier
Loss Function	Mean Absolute Error	Binary Cross-Entropy	Categorical Cross-Entropy
Optimizer	RMSprop	Adam	Adam
Learning Rate	0.001	0.0002	0.001
Epoch	50	50	100
Batch Size	128	128	1024
Validation Split	None	0.1	0.1
Dropout Rate	None	0.4	0.5
Trainable Parameters	219,649	559,617	2,139,486

## Data Availability

Data are contained within the article.

## Abbreviations

The following abbreviations are used in this manuscript:

WPLA	Wireless Physical Layer Authentication
GAN	Generative Adversarial Network
CNN	Convolutional Neural Network
CSI	Channel State Information
CIR	Channel Impulse Response
CFR	Channel Frequency Response
